# Methotrexate-Induced Liver Injury Is Associated with Oxidative Stress, Impaired Mitochondrial Respiration, and Endoplasmic Reticulum Stress In Vitro

**DOI:** 10.3390/ijms232315116

**Published:** 2022-12-01

**Authors:** Saskia Schmidt, Catherine Jane Messner, Carine Gaiser, Carina Hämmerli, Laura Suter-Dick

**Affiliations:** 1 School of Life Sciences, University of Applied Sciences and Arts Northwestern Switzerland, 4132 Muttenz, Switzerland; 2 Department of Pharmaceutical Sciences, University of Basel, 4056 Basel, Switzerland; 3 Swiss Centre for Applied Human Toxicology (SCAHT), 4055 Basel, Switzerland

**Keywords:** methotrexate, liver fibrosis, in vitro model, HepaRG, oxidative stress, stellate cells, mitochondria, ER stress

## Abstract

Low-dose methotrexate (MTX) is a standard therapy for rheumatoid arthritis due to its low cost and efficacy. Despite these benefits, MTX has been reported to cause chronic drug-induced liver injury, namely liver fibrosis. The hallmark of liver fibrosis is excessive scarring of liver tissue, triggered by hepatocellular injury and subsequent activation of hepatic stellate cells (HSCs). However, little is known about the precise mechanisms through which MTX causes hepatocellular damage and activates HSCs. Here, we investigated the mechanisms leading to hepatocyte injury in HepaRG and used immortalized stellate cells (hTERT-HSC) to elucidate the mechanisms leading to HSC activation by exposing mono- and co-cultures of HepaRG and hTERT-HSC to MTX. The results showed that at least two mechanisms are involved in MTX-induced toxicity in HepaRG: (i) oxidative stress through depletion of glutathione (GSH) and (ii) impairment of cellular respiration in a GSH-independent manner. Furthermore, we measured increased levels of endoplasmic reticulum (ER) stress in activated HSC following MTX treatment. In conclusion, we established a human-relevant in vitro model to gain mechanistical insights into MTX-induced hepatotoxicity, linked oxidative stress in HepaRG to a GSH-dependent and -independent pathway, and hypothesize that not only oxidative stress in hepatocytes but also ER stress in HSCs contribute to MTX-induced activation of HSCs.

## 1. Introduction

Drug-induced liver injury (DILI) is a major reason for drug withdrawal and discontinuation of treatment regimens [1,2]. A prominent example of chronic DILI is the folate antagonist methotrexate (MTX) [3]. MTX has been on the market for approximately 70 years as a high-dose treatment (≥500 mg/m^2^) for cancer and a low-dose treatment (5–25 mg/week) for autoimmune diseases, such as psoriasis or rheumatoid arthritis (RA) [3,4,5,6,7]. Low-dose MTX treatment has been linked to several cases of hepatotoxicity in patients [8,9]. A comparative study of 108 patients taking MTX as an RA treatment showed that 29 patients had higher aspartate transaminase (AST) and alanine transaminase (ALT) levels and increased liver stiffness [10]. High levels of AST, ALT, and liver stiffness are early markers for the development of liver fibrosis. Liver fibrosis is characterized by extracellular matrix (ECM) accumulation, resulting in distorted hepatic architecture through fibrous scar formation. The propagation of liver fibrosis is depicted in a so-called adverse outcome pathway (AOP) featuring a series of key events: hepatocyte death/injury leads to Kupffer cell activation, which subsequently releases the pro-fibrogenic cytokine transforming growth factor beta 1 (TGF-β1) to activate hepatic stellate cells (HSCs) [11]. HSCs are the key drivers of ECM deposition and, thus, responsible for the excessive scarring of the liver tissue. Untreated liver fibrosis ultimately progresses into liver cirrhosis and can result in hepatic insufficiency, portal hypertension, and potentially liver failure. Nowadays, the risk of developing hepatic fibrosis and cirrhosis is low when using MTX as a treatment for RA or for psoriasis, as risk factors for hepatotoxicity are taken into account before prescribing MTX and treated patients are closely monitored. However, liver fibrosis is still a potential consequence of the treatment [12,13,14].

In order to investigate the hepatotoxic effects of MTX, its mode of action must be understood. MTX enters the cell, like folinic acid, through the reduced folate carrier 1 (RFC1/SLC19A1) [15]. Intracellularly, it is polyglutamated by folylpolyglutamate synthase (FPGS) to its active form, MTX polyglutamates (MTXPGs). MTXPGs competitively inhibit the dihydrofolate reductase (DHFR), which catalyzes different conversion steps of folate coenzymes essential for the biosynthesis of RNA and DNA [16]. The inhibition of DHFR leads to a cell cycle arrest in the S-phase and, thus, to acute toxicity in fast-dividing and growing cells, such as cancer cells [17]. This mechanism is further enhanced by the inhibition of 5-aminoimidazole-4-carboxamide ribonucleotide (AICAR) transformylase (ATIC) and thymidylate synthase (TYMS) by MTXPGs, which also diminishes the production of purines and pyrimidines. The inhibition of ATIC additionally leads to an increased extracellular accumulation of adenosine. Adenosine functions as an anti-inflammatory agent for immune cells, supporting the anti-inflammatory properties of MTX [18,19,20]. Inflammation is furthermore suppressed by the inhibition of nuclear factor κB (NF-κB) through the increased adenosine release and the decreased reduction of dihydrobiopterin (BH2) to tetrahydrobiopterin (BH4) [15]. 

Several studies have been conducted in patients, animals, and in vitro to investigate the different pathways which could be linked to MTX hepatotoxicity. It has been shown that retention and accumulation of MTXPGs and consequent depletion of folic acid in liver cells leads to an elevation of liver enzyme levels, which might be partly responsible for MTX-induced hepatotoxicity [19]. Pre-treatment of Wistar rats with the isoquinoline alkaloid berberine reduced MTX-induced ALT and AST elevation, replenished the natural antioxidant glutathione (GSH), and restored the activity of glutathione peroxidase (GPx), catalase (CAT), and superoxide dismutase (SOD), which are enzymatic free radical scavengers [21]. Mice pre-treated with inulin additionally showed reduced gene expression levels of pro-apoptotic caspase-3 and miR-122, a marker for hepatocellular damage [22]. In the human liver cell line L-02, fewer apoptotic cells were detected when MTX was co-administered with rhein, an anthraquinone compound. Furthermore, rhein was found to counteract the downregulation of the nuclear factor erythroid 2-related factor 2/heme oxygenase 1 (Nrf2/HO-1) signaling pathway by MTX, which is an essential pathway regulating numerous cytoprotective genes [23]. Taken together, these studies suggest that elevated liver enzyme levels, inhibition of antioxidant pathways, and especially oxidative stress are the main reasons for the development of liver fibrosis triggered by MTX [24,25,26,27,28]. Oxidative stress is the result of an imbalanced redox state: excessive production of pro-oxidants (e.g., reactive oxygen species (ROS)) or exhaustion of antioxidant defenses (e.g., GSH) leads to cellular damage. This hepatocellular damage plays an important role in liver fibrosis AOP [11]. 

Despite the extensive research reports on MTX, little is known about the precise mechanisms by which MTX-induced oxidative stress in hepatocytes leads to the activation of HSCs. Here, we set out to elucidate the mechanisms underlying the hepatotoxic properties of MTX. We used the hepatoma cell line HepaRG and immortalized hepatic stellate cells hTERT-HSC to discern cellular effects. HepaRGs are widely used for in vitro assays in toxicology and metabolism studies due to their suitable characteristics, including cytochrome p450 expression [29,30]. hTERT-HSCs have been shown to respond in a similar manner to primary HSCs with regard to HSC activation [31,32]. In the present study, we investigated the effects of MTX on HepaRG and hTERT-HSCs, alone and as a co-culture. Hepatocellular injury is considered a key event that precedes HSC activation in the development of liver fibrosis [33]. Hence, we focused on MTX-induced injury and increased oxidative stress in the HepaRG monoculture by measuring oxygen consumption rate, superoxide formation, and cell viability. In addition, we assessed the effect of MTX on HSC activation markers. We successfully showed that HepaRGs are sensitive to MTX-induced toxicity. The data suggest that MTX not only induces oxidative stress but also sensitizes cells toward oxidative stress. Furthermore, we propose that the activation of HSC by MTX may be linked to ER stress.

## 2. Results

### 2.1. MTX Causes a Slight Reduction in the Viability of HepaRG at Therapeutic-Relevant Concentrations

**Figure 1:**
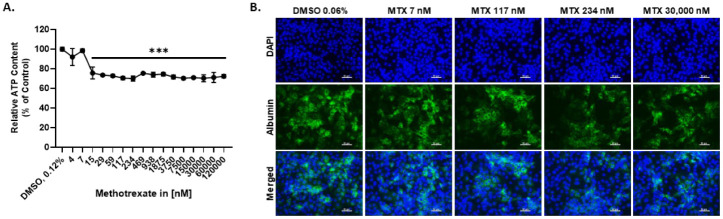
The effect of MTX treatment on HepaRG viability and functionality. HepaRG were exposed to MTX for 72 h. Viability was assessed using the CellTiter-Glo^®^ Luminescent Cell Viability Assay and expressed as relative ATP content (% of control), N = 6–9 (**A**). The HepaRG were fixed and stained for albumin (green) and counterstained with DAPI (blue). Images were taken using the Zeiss Colibri 7 LED system (**B**). Scale bar representing 50 µm. Graphs represent means ± SD; statistical analysis based on one-way ANOVA; ***, *p* ≤ 0.001.

To determine adequate concentrations for MTX, HepaRGs were exposed to a wide range of MTX concentrations (4–120,000 nM) for 72 h, and the cell viability was evaluated by measuring the intracellular ATP content. The results demonstrated that MTX does not elicit a concentration-dependent decrease in viability. Exposure to 4 and 7 nM MTX elicited a 10% decrease in viability, whereas all the other concentrations resulted in a 15–23% decrease (Figure 1A). Concentrations of 7 nM, 117 nM, 234 nM, and 30,000 nM MTX were chosen for all subsequent experiments to ensure that the cell viability was always between 75% and 90%. Concomitantly to the decrease in cellular ATP levels, MTX led to an apparent reduction of the expression of albumin, assessed by immunohistochemistry (IHC) (Figure 1B and Figure S1).

### 2.2. HepaRG Express Folate Transporter SLC19A1 and DHFR

**Figure 2:**
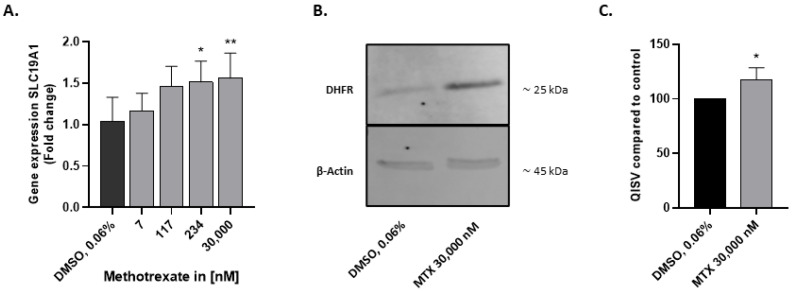
Expression of folate transporter SLC19A1 and DHFR in HepaRG. HepaRG were treated as previously described with or without MTX for 72 h. Gene expression of folate transporter SLC19A1, responsible for intracellular uptake of MTX, was detected using q-RT-PCR. Data are expressed as fold change, N = 3 (**A**). HepaRG were treated with 30,000 nM MTX for 7 days and processed for Western blot analysis. Anti-DHFR antibody was used to detect DHFR, β-actin was used as loading control (**B**). Semi-quantification of DHFR normalized to β-actin band intensity, N = 3 (**C**). Quantitative intensity staining value (QISV); Bar graphs represent means ± SD; statistical analysis based on one-way ANOVA (**A**) and Student’s unpaired *t*-test (**C**); *, *p* ≤ 0.05; **, *p* ≤ 0.01.

For the development of a suitable in vitro model to study MTX toxicity, we first verified the expression of the folate transporter SLC19A1 and the enzymatic target of MTX (DHFR) in HepaRG. Cells were exposed to a range of MTX concentrations for 3 days, and RNA was extracted for q-RT-PCR analysis. SLC19A1 was expressed in HepaRG, and its expression was slightly upregulated by the treatment (Figure 2A). To investigate DHFR expression, HepaRG were exposed to 30,000 nM MTX for 7 days, followed by a total protein extraction. DHFR was detected in the protein fraction by Western blot analysis (Figure 2B). Quantification of the detected bands revealed a higher abundance of DHFR in HepaRG exposed to MTX (Figure 2C).

### 2.3. N-Acetylcysteine Alleviates Superoxide Formation Induced by MTX in HepaRG

**Figure 3:**
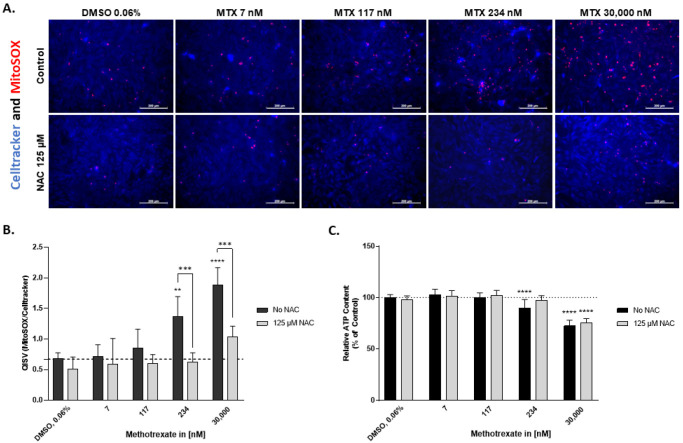
NAC treatment reduces superoxide formation in MTX-treated HepaRG. HepaRG were pre-exposed for 2 h to 125 µM NAC or left untreated, then exposed to MTX without NAC or MTX with NAC for 72 h. The HepaRG were stained live with Celltracker violet BMQC (blue) and MitoSOX^TM^ (red) to investigate superoxide production following MTX exposure with and without NAC. Images were taken using the Zeiss Colibri 7 LED Fluorescence system (**A**). Scale bar representing 200 µm. The quantity of the MitoSOX^TM^ was measured using ImageJ and normalized to the quantity of Celltracker violet BMQC. Data are expressed as QISV, N = 3 (**B**). Viability was assessed using the CellTiter-Glo^®^ Luminescent Cell Viability Assay and expressed as relative ATP content (% of control), N = 3 (**C**). For better illustration, dashed lines have been added showing the mean value of the solvent control DMSO, 0.06% (**B**,**C**). Bar graphs represent means ± SD; statistical analysis based on two-way ANOVA; **, *p* ≤ 0.01; ***, *p* ≤ 0.001; ****, *p* ≤ 0.0001.

The reduced viability in HepaRG exposed to MTX could be a result of oxidative stress. Thus, HepaRG were stained with Celltracker and MitoSOX^TM^ to investigate superoxide formation in mitochondria after exposure to varying concentrations of MTX for 72 h with or without pre-incubation with the antioxidant N-Acetylcysteine (NAC). NAC was added to replenish the intracellular GSH stocks and to increase the cellular tolerance to oxidative stress. Without NAC, the cells showed a significant concentration-dependent increase in superoxide formation above 117 nM MTX compared to the untreated control (Figure 3A). The addition of NAC reduced the formation of superoxide (Figure 3B). Measuring the total intracellular GSH levels confirmed that NAC elevates the total GSH concentration. MTX, on the other hand, decreased the GSH content (Figure S2). Pre-incubation with NAC rescued MTX-induced reduction in HepaRG viability below 234 nM treatment. For 30,000 nM MTX, NAC failed to rescue the cell viability (Figure 3C). Altogether, the data show that MTX induced the formation of superoxide in HepaRG, which was ameliorated by a pre-incubation with NAC.

### 2.4. Spare Respiratory Capacity Is Reduced in HepaRG Exposed to MTX

**Figure 4:**
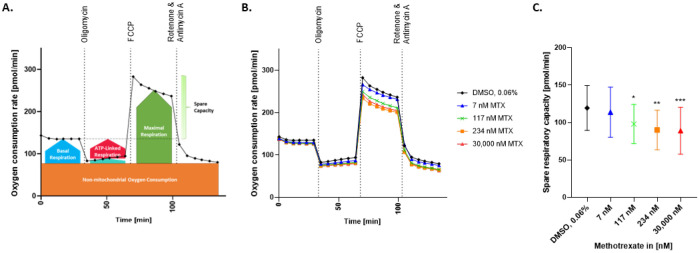
Measurement of the oxygen consumption rate in MTX-treated HepaRG. Mito Stress test profile (**A**). The oxygen consumption rate (OCR) of HepaRG exposed to MTX for 72 h was measured using the Seahorse XF96 analyzer (**B**). The HepaRG were sequentially exposed to three treatments: Oligomycin (1 μM), FCCP (2.5 μM), and a mix of Rotenone (0.5 μM) + Antimycin A (0.5 μM). OCR was measured 6 times per treatment step and expressed as pmol/min, N = 3 (**B**). Spare respiratory capacity was calculated for each condition as the difference from the basal to the maximal OCR (**C**). Graphs represent means (**B**) or means ± SD (**C**); statistical analysis based on one-way ANOVA; *, *p* ≤ 0.05; **, *p* ≤ 0.01; ***, *p* ≤ 0.001.

As a measure of mitochondrial respiration and resistance of cells to stress, maximal oxygen consumption rate (OCR) and the spare respiratory capacity (SRC) were assessed using the Seahorse XF96 analyzer (Figure 4A). After treating HepaRG with different concentrations of MTX for 3 days, a concentration-dependent decrease in the maximal OCR and SRC was observed (Figure 4B,C).

**Figure 5:**
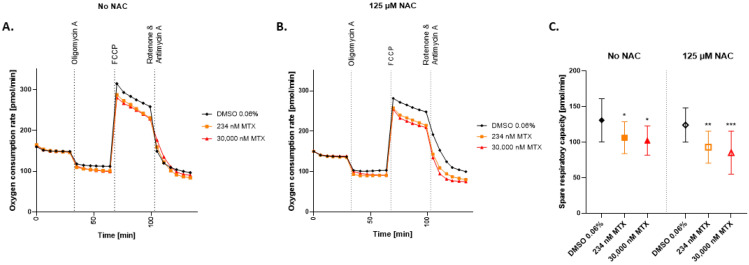
Effect of NAC on oxygen consumption rate in MTX treated HepaRG. The oxygen consumption rate (OCR) of HepaRG exposed to MTX for 72 h (**A**) or HepaRG preincubated with 125 μM NAC and then exposed to MTX for 72 h (**B**), was measured using the Seahorse XF96 analyzer. The HepaRG were sequentially exposed to three treatments: Oligomycin (1 μM), FCCP (2.5 μM), and a mix of Rotenone (0.5 μM) + Antimycin A (0.5 μM). OCR was measured 6 times per treatment step and expressed as pmol/min. Spare respiratory capacity was calculated as the delta maximal respiration to basal respiration (**C**). Graphs represent means (**A**,**B**), and means ± SD (**C**); statistical analysis based on two-way ANOVA; *, *p* ≤ 0.05; **, *p* ≤ 0.01; ***, *p* ≤ 0.001.; N = 3.

Interestingly, the effects on mitochondrial respiration could not be prevented by incubation with NAC. As depicted in Figure 5, pre-treatment of the cells with NAC (125 μM) did not prevent the effect of MTX on the OCR and SRC at 234 nM and 30,000 nM MTX.

### 2.5. 2D hTERT-HSC Are Activated by MTX Treatment in Mono- and Co-Cultures with HepaRG

**Figure 6:**
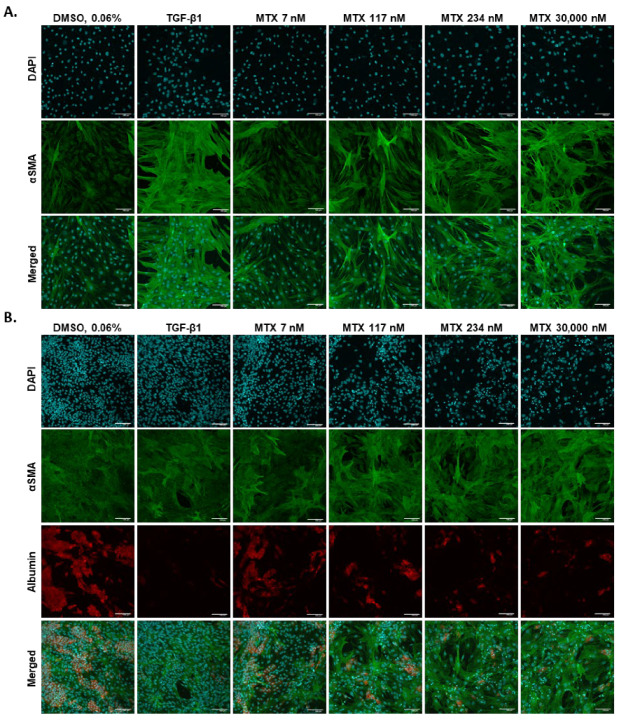
MTX triggers the activation of 2D hTERT-HSC in mono- and co-culture with HepaRG. Monocultures of 2D hTERT-HSC (**A**), or co-cultures with hTERT-HSC and HepaRG (**B**), were exposed to varying concentrations of MTX or the positive control TGF-β1 for 7 days. Cells were stained for stress marker αSMA (green), hepatocyte marker albumin (red), and counterstained with DAPI (blue). Scale bar representing 100 µm.

Activated HSCs are the key drivers of liver fibrosis progression. To see whether MTX activates hTERT-HSC in 2D, monocultures of hTERT-HSC were exposed to MTX or the positive control TGF-β1 for 7 days. The cells were fixed and stained for the activation marker alpha-smooth muscle actin (αSMA). Upon exposure to TGF-β1, hTERT-HSC underwent activation, shown by an elongated shape and an increase in the expression of αSMA. Similarly, MTX elicited a concentration-dependent increase in αSMA stress fibers above 117 nM (Figure 6A and Figure S3A).

To elucidate the interplay between hepatocytes and HSC, co-cultures of HepaRG and hTERT-HSCs were treated as described for the monoculture. In addition to the αSMA staining, HepaRG were stained for the hepatocyte marker albumin to distinguish between the different cell types. Treatment with TGF-β1 led to depletion of albumin in the HepaRG, whereas in the hTERT-HSC, it triggered activation represented by an increased expression of stress fibers. Exposure to MTX led to a concentration-dependent decrease in albumin and an increase in αSMA above 117 nM MTX (Figure 6B and Figure S3B,C). For both the mono- and co-cultures, a fibrotic phenotype was established after 7 days of treatment with MTX or TGF-β1.

### 2.6. MTX-Induced ER-Stress as a Possible Activator of hTERT-HSC

**Figure 7:**
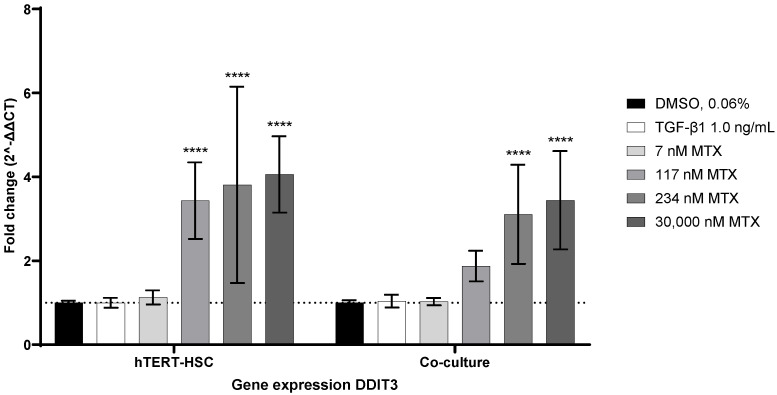
MTX treatment induces gene expression of ER-stress marker DDIT3 in mono- and co-cultures. hTERT-HSC monocultures or co-cultures with HepaRG were exposed to different MTX concentrations or TGF-β1 for 7 days. Q-RT-PCR was performed to detect expression levels of DDIT3. For a better visual representation, the dashed line showing the baseline expression of DDIT3 in the solvent control, was added. Bar graphs represent means ± SD; statistical analysis based on one-way ANOVA; ****, *p* ≤ 0.0001; N = 3.

To investigate whether endoplasmic reticulum (ER) stress triggered by MTX could be involved in the activation of HSC, monocultures of hTERT-HSC and co-cultures of hTERT-HSC and HepaRG were treated with different MTX concentrations or with TGF-β1 for 7 days. mRNA expression of C/EBP homologous protein (CHOP, encoded by DDIT3) was analyzed as the main actor in the PKR-like ER kinase (PERK)/CHOP pathway. Treatment with TGF-β1 did not affect the expression of DDIT3. Exposure to MTX, on the other hand, increased the expression of DDIT3, starting from 117 nM in a concentration-dependent manner in both the mono- and co-culture (Figure 7). Thus, MTX but not TGF-β1 triggered ER stress via the PERK/CHOP pathway in the mono- and co-cultures.

## 3. Discussion

The disease-modifying anti-rheumatic drug MTX is still the gold standard for the therapy of RA, despite its known adverse events, such as hepatotoxicity and nephrotoxicity [15,34]. The mechanism by which MTX induces fibrosis in low-dose treatment regimens is yet to be elucidated. In this study, we used the human hepatic cell line HepaRG, which closely mimics the metabolic activity and gene expression of primary human hepatocytes, and the immortalized cell line hTERT-HSC as a surrogate for HSC, to further investigate the hepatotoxic properties of MTX. Firstly, we established that HepaRG cells express the folate transporter (SLC19A1) and the drug target (DHFR) necessary to allow a pharmacological effect. We determined that therapeutic concentrations of MTX lead to superoxide formation and impairment of mitochondrial respiration. We were also able to demonstrate that hTERT-HSC in co-culture with HepaRG undergo activation after sub-chronic treatment with MTX for 7 days. We propose a close interplay of oxidative stress in HepaRG and ER stress in hTERT-HSC as the drivers of hTERT-HSC activation and, thus, for the propagation of liver fibrosis.

Treatments with MTX in humans range from 10 mg/m^2^–12 g/m^2^, with peak serum concentrations from 0.5 µM to 1000 µM [35,36,37,38,39]. Since we focused on low-dose MTX toxicity, our chosen concentrations to assess viability in HepaRG ranged from 4 nM–120,000 nM, well within the clinical systemic range. The decrease in cell viability plateauing at around 20% toxicity across a wide range of concentrations confirmed the broad therapeutic window of MTX. Similar responses were seen in 2D HepG2, HeLa, and HEK293 cells [40,41,42]. In 2D HepaRG, 100–150,000 nM MTX induced the accumulation of neutral lipids and reduced the viability to 70% [43]. Previous results from our laboratory demonstrated that ten days of exposure to 30,000 nM MTX induced fibrosis in a 3D in vitro model using HepaRG, hTERT-HSC, and THP-1, thus we set 30,000 nM MTX as the upper treatment limit [44]. For the lower treatment limit, we used 7 nM, a subtoxic concentration. To investigate the turning point from subtoxic to toxic concentrations, we chose 117 and 234 nM MTX. Consistent with observations in patients and in vivo, albumin levels were only slightly reduced in the in vitro HepaRG model [45,46].

The intracellular uptake of MTX by SLC19A1 is a major determinant for the effectiveness of MTX treatment, and it is crucial that an in vitro cell model to study the effect of MTX expresses this uptake transporter. Genetic variants and depletion of this transporter result in drug resistance or ineffectiveness of the treatment [47,48,49,50]. RNA sequencing of HepaRG revealed the expression of SLC19A1 [51]. The impact of MTX on the expression of SLC19A1 in HepaRG has, however, not yet been investigated. Transcriptional analysis using qPCR confirmed the expression of SLC19A1 in HepaRG. Moreover, this transporter appeared upregulated by the treatment, indicating that MTX is able to enter the HepaRG cells. This induction was also seen in studies with a breast cancer cell line and laryngeal cancer cells [52,53]. Similarly, we could demonstrate by Western blot analysis that the drug target DHFR is present in HepaRG and that this enzyme seems more abundant in MTX-treated cells. This has also been shown in the leucocytes of patients treated for leukemia [54,55]. MTX regulates the expression of DHFR on a translational level. Under normal conditions, DHFR either binds to its co-factor nicotinamide adenine dinucleotide phosphate (NADPH) or its cognate mRNA, which hinders the synthesis of DHFR. These two conformers are in equilibrium. Competitive binding of MTX to DHFR leads to the dissociation of the DHFR–mRNA complex, resulting in a resumed translation of DHFR and, thus, increased protein levels [56]. In RA, increased levels of DHFR have not been linked to MTX resistance, whereas for the treatment of osteosarcoma, upregulation of DHFR is involved in drug resistance [57,58,59]. Our results regarding low-level toxicity, expression of SLC19A1 transporter, and upregulation of DHFR protein levels strongly suggest that MTX is reaching its pharmacological target in the in vitro model. The pharmacological activity of MTX may be intimately related to the hepatotoxicity, as inhibition of DHFR by MTXPGs does not only cause desired effects, such as depletion of RNA and DNA building blocks, but it also hinders the reduction of BH2 to BH4, which causes an increase in the production of ROS [15].

Our results confirm that low concentrations of MTX cause oxidative stress in HepaRG cells, which was determined by the significantly increased formation of superoxide. The MTX-elicited accumulation of superoxide in the mitochondria could be prevented by supplying the cells with NAC, a precursor for glutathione synthesis. In mitochondria, there is a high abundance of GSH, which is particularly important for the disposal of hydrogen peroxide generated from superoxide anion. Depletion of this mitochondrial GSH (mGSH) has been linked to hepatic disease progression, such as non-alcoholic liver disease and cholestasis [60]. As previously published, MTX downregulates the Nrf2/HO-1 axis and hence, inhibits the synthesis of mGSH [23]. By pre-incubation of the cells with NAC, superoxide concentration drastically dropped, as was expected based on the role of GSH in antioxidant defense [61,62]. The effects of NAC were also confirmed by an improvement in cell viability below 234 nM MTX. Toxicity upon treatment with 30,000 nM MTX could only partly be reversed by NAC.

To further investigate the impact of MTX on mitochondria, we assessed cellular respiration by performing a Mito Stress Test using the Seahorse XF96 Analyzer. Quantification of cellular respiration is a direct indicator of mitochondrial function as it detects electron transport chain (ETC) impairment and is highly dependent on consecutive reactions from glycolysis to oxidative phosphorylation [63]. The Mito Stress Test targets different complexes in the ETC to measure ATP-linked respiration, proton leak, maximal respiration, spare respiratory capacity (SRC), and non-mitochondrial oxygen consumption [64]. The SRC is a robust parameter to evaluate mitochondrial fitness, where high levels of SRC represent healthy mitochondria, and low levels of SRC correspond to mitochondrial dysfunction [65]. In our hands, we could show a significant decrease in SRC already at 117 nM MTX, in concordance with the low dose effects that MTX has on HepaRG, as described above. Our findings further support the key role of mitochondrial stress in hepatocellular toxicity by MTX. Previous publications using HepG2 cells also reported a decrease in SRC by MTX but at much higher concentrations (3 mM) [42]. Having shown the antioxidant effects of NAC in the MitoSOX^TM^ assay, we tested whether pre-incubation with NAC could also reverse the effects of MTX on OCR and SRC. Pre-incubation of HepaRGs with NAC before exposure to MTX did not, however, improve the maximal respiration nor the SRC. Taking into consideration that accumulation of H_2_O_2_ could be prevented by boosting the GSH concentration, but the impairment of mitochondrial respiration could not, we conclude that at least two phenomena are involved in the toxicity of HepaRG exposed to MTX: (i) MTX causes oxidative stress and depletes GSH and (ii) MTX affects the cellular respiration in a GSH-independent manner. The underlying mechanism of MTX-hepatotoxicity in a chronic setting may result from the classical superoxide formation that consumes mGSH stocks and from the downregulation of the Nrf2/HO-1 pathway additionally inhibiting the synthesis of new GSH, leading to a depletion of antioxidant defense mechanisms and higher ROS levels. An additional GSH-independent pathway seems to affect cellular respiration, sensitize the mitochondria to oxidative stress, and reduce their ability to cope with increased energy demand.

In addition to the moderate but consistent toxicity towards HepaRG, we investigated the effects of MTX on the hepatic stellate cell. Progression of fibrosis is closely linked to the activation of HSCs and subsequent deposition of ECM into the space of Disse, leading to increased liver stiffness and portal hypertension [66]. Several pathways are involved in the activation of HSCs, including oxidative stress, ER stress, and activation of the A_2A_ receptor by adenosine [67,68]. Exposure of hTERT-HSC to MTX resulted in a concentration-dependent increase in stress fibers, representing an activated phenotype of HSC. This direct activation of HSC by MTX in 2D has also been reported in LX-2 (100 nM) and primary HSC (1000 nM) [69,70]. In addition to the activation of HSC by pro-fibrotic compounds, the release of ROS and cytokines by injured hepatocytes and the engulfment of apoptotic hepatocyte bodies by HSC further enhances the process of activation [11]. To test whether MTX-induced oxidative stress in HepaRG triggers activation in hTERT-HSC, we exposed our co-cultures to MTX. A concentration-dependent increase in the stress marker αSMA in hTERT-HSC and a decrease in albumin expression in HepaRG confirmed a fibrotic phenotype in the co-culture model. Interestingly, the reduced expression of albumin in the co-culture was more pronounced than in the HepaRG monoculture. This observation undermines the importance of cellular crosstalk in the progression of fibrosis and also for the development of a reliable and physiological in vitro model. In addition to oxidative stress, ER stress has recently been linked to MTX-induced nephrotoxicity and also to HSC activation in patient samples, LX-2 cells, primary HSC, and mice [71,72]. HSCs are rich in ER and, thus, sensitive to ER stress. ER stress can be counteracted by activation of the unfolded protein response (UPR), which acts via three branch pathways [73]. One of which is the PKR-like ER kinase (PERK) pathway. Under stress conditions, PERK is activated by the dissociation of the ER chaperon BiP, leading to downstream activation of CHOP (encoded by DDIT3), an inducer of apoptosis. Fredriksson et al. investigated the interrelation of oxidative stress, ER stress, and translational regulation in compounds causing DILI [74]. For the majority of the compounds investigated, upregulation of the Nrf2-related oxidative stress response and activation of the translation initiation signaling pathway in combination with ER stress responses, were identified as the most important cell toxicity pathways in HepG2. MTX on the other hand, induced ER stress primarily through the PERK/CHOP pathway, independent of the Nrf2 antioxidant signaling pathway. Additionally, in a study by Koo et al., activation of the PERK/CHOP pathway in LX-2 cells and primary rat HSCs was shown to trigger the destabilization of the heterogeneous nuclear ribonucleoprotein A1 (HNRN-PA1), which facilitates the dysregulation of miR-18A [71]. miR-18A is an inhibitor of SMAD2, and its depletion has been linked to an increased expression of αSMA. Overall, ER stress-induced SMAD2 overexpression via the PERK/CHOP pathway resulted in an increased expression of pro-fibrotic genes, including αSMA, collagen 1A1, and connective tissue growth factor [75]. Thus, we focused on DDIT3 as a marker for MTX-induced ER stress. In the hTERT-HSC monoculture and co-cultures with HepaRG, ER stress, represented by a significant upregulation of DDIT3 above 117 nM MTX, was detected. Treatment with TGF-β1, the positive control for stellate cell activation, had no effect on the gene expression of DDIT3. Similarly, a recent study by Yang et al. showed that exposure of lung resident mesenchymal/stromal cells (LR-MSC) to TGF-β1 and subsequent differentiation of the LR-MSC to myofibroblasts did not affect the expression of DDIT3 [75]. However, overexpression of CHOP facilitated TGF-β1-induced myofibroblast transformation of LR-MSC. TGF-β1 promotes stellate cell activation via two main pathways: the SMAD2/3 (canonical) and the mitogen-activated protein kinase signaling (MAPK, noncanonical) pathway [76]. Thus, the facilitated myofibroblast transformation seen in LR-MSC could be the result of synergistic activation of SMAD2 by CHOP and TGF-β1. Taking the increased protein levels of αSMA and the increased mRNA levels of DDIT3 together, we hypothesize that ER stress may contribute to MTX-induced activation of HSC via the TGF-β/SMAD pathway.

In conclusion, we showed that HepaRGs are a pharmacologically relevant in vitro model to assess MTX-induced hepatotoxicity. Additionally, we were able to link oxidative stress caused by MTX to a GSH-dependent and -independent pathway: (i) depletion of GSH resulting in an increase of ROS, and (ii) impairment of the SRC sensitizing mitochondria to oxidative stress. We further conclude that not only oxidative stress in HepaRG but also ER stress in HSC may play a major role in the MTX-induced activation of HSC.

## 4. Materials and Methods

### 4.1. Human Cell Lines and Cell Seeding

All cells were cultured at 37 °C in 5% CO_2_ in a humified incubator and passaged using Trypsin-EDTA (Sigma, Taufkirchen, Germany, 59417C-100ML). HepaRG cells (Biopredic International, Saint Grégoire, France, HPR101) were seeded at 1 × 10^5^ undifferentiated cells/cm^2^ in basal medium (Biopredic International, Saint Grégoire, France, MIL700C) with growth supplements ADD710C (Biopredic International, Saint Grégoire, France). After 14 days of culture, cell medium was exchanged to medium containing 50:50 growth and differentiation supplements ADD720C (Biopredic International, Saint Grégoire, France) for 4 days and then exchanged to only differentiation supplements for a further 10 days prior to use. HepaRG were used at passages below 20. Differentiated HepaRG were seeded for experimentation in 96-well plates at 6.25 × 10^4^ cells/cm^2^ in differentiation medium for 3–10 days prior to treatment. Treatment medium: William’s E Medium supplemented with GlutaMAX (ThermoFisherScientific, Reinach, Switzerland, 32551087), 1× ITS (Sigma, Taufkirchen, Germany, 11074547001), 100 nM Dexamethasone (Sigma, Taufkirchen, Germany, D1756) and 1% penicillin and streptomycin (P/S) (Sigma, Taufkirchen, Germany, P4333-100ML).

hTERT-HSC were kindly provided by Dr. Bernd Schnabl (UC, San Diego, CA 92103, USA). hTERT-HSC were cultured in Dulbecco′s Modified Eagle′s Medium (DMEM) High Glucose (ThermoFisherScientific, Reinach, Switzerland, 41965062) supplemented with 10% fetal bovine serum (FBS; FisherScientific, Reinach, Switzerland, 10270-106) and 1% P/S. hTERT-HSC were used at low passages (<12) to avoid spontaneous activation. hTERT-HSC were seeded in 96-well plates at 1.25 × 10^4^ cells/cm^2^ and allowed to adhere overnight prior to treatment.

Co-culture experiments were seeded sequentially. HepaRG were seeded first at 6.25 × 10^4^ cells/cm^2^ in differentiation medium in a 96-well plate for 2–9 days, followed by the hTERT-HSC in DMEM with supplements at 1.25 × 10^4^ cells/cm^2^ 24 h prior to treatment.

### 4.2. Cell Treatments

HepaRG, hTERT-HSCs, or the HepaRG-hTERT-HSC co-cultures were seeded as described above, and treatment was carried out using an FBS-free treatment medium, which was refreshed every 2–3 days. The cells were treated with methotrexate (Sigma, Taufkirchen, Germany, M8407-100MG) using the concentration range of 4–120,000 nM for 3–7 days. In addition, TGF-β1 (Sigma, Taufkirchen, Germany, T5050-1UG) 1 ng/mL, which is known to cause hepatocellular damage and to elicit a pro-fibrotic effect, was used as a positive control for hTERT-HSC activation.

N-acetylcysteine (Sigma, Taufkirchen, Germany, A8199) was used as an antioxidant compound. A 2 h pre-incubation was carried out using 125 µM NAC at 37 °C in 5% CO_2_. Following the pre-incubation, the NAC treatment was refreshed and added with the MTX treatments and the untreated medium for a 72 h exposure period.

### 4.3. Cell Viability Assays

Cell viability of HepaRG was assessed using the CellTiter-Glo^®^ Luminescent Cell Viability Assay (Promega, Dübendorf, Switzerland, G7571) after 3 days of MTX treatment. The assay was carried out as described in the manufacturer’s protocol, and luminescence was measured on a Flexstation 3 microplate reader (Bucher Biotec, Basel, Switzerland, MDC Flexstation III S/N FV-01043) at 1000 ms.

### 4.4. Immunohistochemistry

If not stated otherwise, compounds were obtained from Sigma (Taufkirchen, Germany). Cells were washed using PBS containing calcium and magnesium, then fixed with 4% PFA for 15 min. The samples were permeabilized using 0.1% Triton X-100 for 15 min and then blocked with 1% BSA for 1 h at RT. Primary antibodies were diluted as described in Table 1 in 0.1% BSA, and incubation was carried out overnight at 4 °C. Following primary incubation, the samples were washed with PBS and then incubated for 1 h at RT with secondary antibodies diluted as described in Table 1, also in 0.1% BSA. Washing was carried out again, and the samples were counterstained with DAPI for 5 min at RT prior to imaging. Images were taken using the Zeiss Colibri 7 LED system or Olympus Fluoview FV3000 Confocal Microscope.

### 4.5. Gene Expression Analysis

For detection of SLC19A1, HepaRG were treated with MTX for 3 days; for ACTA2 and DDIT3 detection, mono- and co-cultures were exposed to MTX or TGF-β1 for 7 days. mRNA was isolated following standard TRIzol extraction procedure with Glycogen (ThermoFisherScientific, Reinach, Switzerland, LT-02241) from cells lysed using QIAzol Lysis Reagent (Qiagen, Basel, Switzerland, 79306). Reverse transcription was carried out using M-MLV Reverse transcriptase (Promega, Dübendorf, Switzerland, M1705) and oligo dT (Qiagen, Basel, Switzerland, 79237). Real-time PCR was performed using either FastStart TaqMan^®^ Probe Master (Sigma, Taufkirchen, Germany, 4673417001; for SLC19A1) or GoTaq^®^ Probe qPCR and RT-qPCR Systems (Promega, Dübendorf, Switzerland, A6102; for DDIT3 and ACTA2) and TaqMan probes of selected genes (see Table 2). The q-RT-PCR Program used: 10 min denaturation at 95 °C, followed by 40 cycles of 15 s at 95 °C and 1 min at 60 °C. The Ct values were generated using the Corbett Rotorgene Analysis Software 6000 (SLC19A1) or the LightCycler^®^ 480 Systems (DDIT3 and ACTA2) and processed on GraphPad Prism using B2M as the internal standard for normalization. Data are expressed as fold change.

### 4.6. Western Blot Analysis

For Western blot analysis, HepaRG were seeded in a 6-well plate at 2.25 × 10^5^ cells/cm^2^. After 10 days, cells were exposed to 30,000 nM MTX for 7 days. Cells were washed with ice-cold PBS and lysed in Pierce^®^ RIPA Buffer (ThermoFisherScientific, Reinach, Switzerland, 89900) to extract total protein. Protein concentration was determined following the standard Bradford assay protocol using ROTI^®^ Nanoquant (ROTH, Arlesheim, Switzerland, K880.1). Samples were run on a 12.5% SDS-Page and transferred using a wet chamber to pre-activated PVDF membranes (Sigma, Taufkirchen, Germany, IPVH00010). Membranes were blocked for 1.5 h with 4% milk in TBS-T (0.1% Tween-20). After overnight incubation with primary antibody at 4 °C, membranes were washed with TBS-T, and secondary antibody was added for 1 h at RT (see Table 3). Both antibodies were diluted in 3% milk in TBS-T. Membranes were washed again and imaged using the Odyssey^®^ CLx imaging system (LI-COR Biosciences, Bad Homburg, Germany) The quantitative immunostaining value (QISV) was calculated using ImageJ by measuring the area and intensity of DHFR bands and normalizing it to the area of β-actin bands.

### 4.7. MitoSOX^TM^ Red Mitochondrial Superoxide Indicator

After 3 days of MTX treatment, HepaRG were washed with PBS and then incubated with 10 µM Celltracker violet BMQC (ThermoFisherScientific, Reinach, Switzerland, C10094) and 5 µM MitoSOX^TM^ (ThermoFisherScientific, Reinach, Switzerland, M36008) diluted in PBS with Mg^2+^ and Ca^2+^ for 20 min. Following the 20 min incubation, the cells were washed gently 3 times using PBS with Mg^2+^ and Ca^2+^ and 10% FBS and imaged in treatment medium. Live imaging was carried out using a Zeiss Colibri 7 LED system. QISV was calculated using ImageJ by measuring the area and intensity of positive MitoSOX^TM^ staining and normalizing it to the area of Celltracker violet BMQC.

### 4.8. Seahorse XF96 Analyzer

HepaRG were seeded at 6.8 × 10^5^ cells/cm^2^ in an XF96 cell culture microplate (Bucher Biotec, Basel, Switzerland, 101085-004) following cell number titration and allowed to adhere for 72 h. Cells were then treated as described in the cell treatments section. Once the treatment period was finished, the Agilent Seahorse XF96 Extracellular Flux Analyzer was used to assess the OCR of the cells performing a Mito Stress Test. The seahorse was calibrated using XF Calibrant (Bucher Biotec, Basel, Switzerland, 100840-000). The medium was exchanged on the cells to DMEM high glucose supplemented with 2 mM L-Glutamine (Sigma, Taufkirchen, Germany, G7513). The cells were sequentially exposed to three treatments and measured 6 times per treatment step, as described in Table 4. Briefly, prior to modulating the ETC, basal respiration is measured. For measuring the ATP-linked respiration, oligomycin is injected, which inhibits the ATP synthase (complex V) in the membrane of mitochondria and hence prevents all respiration requiring ATP. Following this, FCCP, an uncoupling agent, is injected, which leads to an uninhibited flow of electrons through the ETC and, thus, maximal oxygen consumption by complex IV. The final injection of rotenone, a complex I inhibitor, and antimycin A, a complex III inhibitor, leads to a complete inhibition of mitochondrial respiration. The maximal oxygen consumption after FCCP injection can be used to calculate the SRC, which indicates a cell’s ability to react to a higher energy demand or to acute cellular stress.

### 4.9. Statistical Analysis

Data were analyzed using GraphPad Prism 9 (GraphPad Software, Version 9.3.0) and expressed as mean values ± SD. The Student’s *t*-test was used for comparison between two groups, and one-way ANOVA was used for statistical analysis of multiple concentrations of the same treatment. Two-way ANOVA was used for statistical analysis of multiple concentrations and different treatments. *p* < 0.05 was considered to be significant: *, *p* ≤ 0.05; **, *p* ≤ 0.01; ***, *p* ≤ 0.001; ****, *p* ≤ 0.0001.

## Figures and Tables

**Table 1:** Antibodies used for immunohistochemistry.

Protein of Interest	Primary Antibody	Secondary Antibody
αSMA	Mouse polyclonal antibody (Sigma, Taufkirchen, Germany, A5228)/1:400	Goat anti-mouse Alexafluor 488 (Sigma, Taufkirchen, Germany, A-11017)/1:1000
Albumin	Rabbit monoclonal antibody (Abcam, Cambridge, United Kingdom, ab207327)/1:800	Goat anti-rabbit Alexafluor 546 (Sigma, Taufkirchen, Germany, A-11071)/1:1000

**Table 2:** TaqMan probes of selected genes obtained from ThermoFisherScientific (Reinach, Switzerland).

Gene of Interest	Abbreviation	Ref. Nr
Beta-2-Microglobulin	B2M	Hs00187842_m1
Solute Carrier Family 19 Member 1 (folate transporter 1)	SLC19A1	Hs00953344_m1
DNA Damage Inducible Transcript 3	DDIT3	Hs00358796_g1

**Table 3:** Antibodies for Western blot.

Protein of Interest	Primary Antibody	Secondary Antibody
Dihydrofolate reductase (DHFR)	Rabbit polyclonal antibody (ThermoFisherScientific, Reinach, Switzerland, PA5-90913)/1:400	Donkey anti-rabbit IRDye^®^ 680RD (LI-COR Biosciences, Bad Homburg, Germany, 926-68073)/1:20,000
Beta-actin (β-actin)	Mouse monoclonal antibody (Santa Cruz Biotechnology, Heidelberg, Germany, sc-47778)/1:1000	Goat anti-mouse IRDye^®^ 800CW (LI-COR Biosciences, Bad Homburg, Germany, 926-32210)/1:20,000

**Table 4:** Seahorse treatment specifications.

Condition	Measurement Timeline (min)	Electron Transport Chain Target	Effect on OCR
Basal OCR	0, 6, 12, 17, 23, 29		
Oligomycin 1 µM	35, 41, 47, 52, 58, 64	ATP Synthase (Complex V)	Decrease
Cyanide-4 (trifluoromethoxy) phenylhydrazone (FCCP) 2.5 µM	70, 76, 82, 87, 93, 99	Inner mitochondrial membrane	Increase
Rotenone 0.5 µM + Antimycin A 0.5 µM	105, 111, 117, 123, 128, 134	Complex I and III (respectively)	Decrease

## Data Availability

Data is contained within the supplementary material.

## Supplementary Materials

The following supporting information can be downloaded at: https://www.mdpi.com/article/10.3390/ijms232315116/s1.
