# Supplementary material for: Methotrexate-Induced Liver Injury Is Associated with Oxidative Stress, Impaired Mitochondrial Respiration, and Endoplasmic Reticulum Stress In Vitro
Source: Int J Mol Sci. 2022 Dec 1;23(23):15116. doi: 10.3390/ijms232315116 (PMC9735468; doi:10.3390/ijms232315116)
Supplement: Supplementary file 1 [file ijms-23-15116-s001.zip › ijms-2018946-supplementary_v1.pdf]

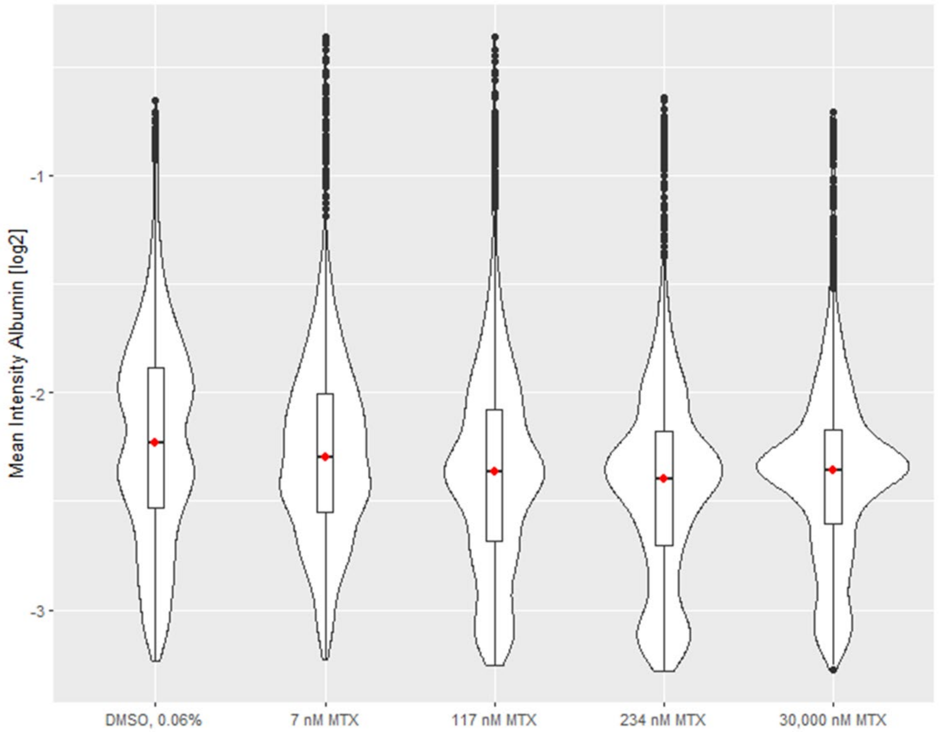

Figure S1. Intensity measurement of albumin expression in HepaRG exposed to MTX. HepaRG were exposed to MTX for 72 h. The HepaRG were fixed and stained for albumin and counterstained with DAPI (Figure 1B). Single cell analysis was performed using CellProfiler and RStudio. Median depicted in red.

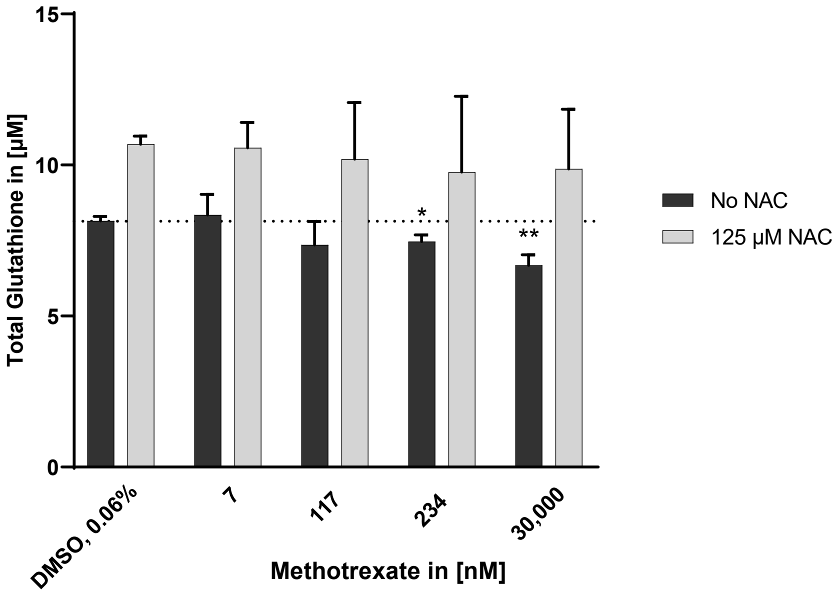

Figure S2. NAC treatment maintains total glutathione levels in HepaRG. HepaRG were pre-exposed for 2 h to 125 µM NAC or left untreated, then exposed to MTX without NAC or MTX with NAC for 72 h. Total glutathione was measured intracellularly using the GSH/GSSG-glo assay® (Promega, Dübendorf, Switzerland, V6612). Data are expressed as total glutathione content in µM, N = 1. Bar graphs represent means ± SD; statistical analysis based on Two-way ANOVA; *, $p \leq 0.05$; **, $p \leq 0.01$.

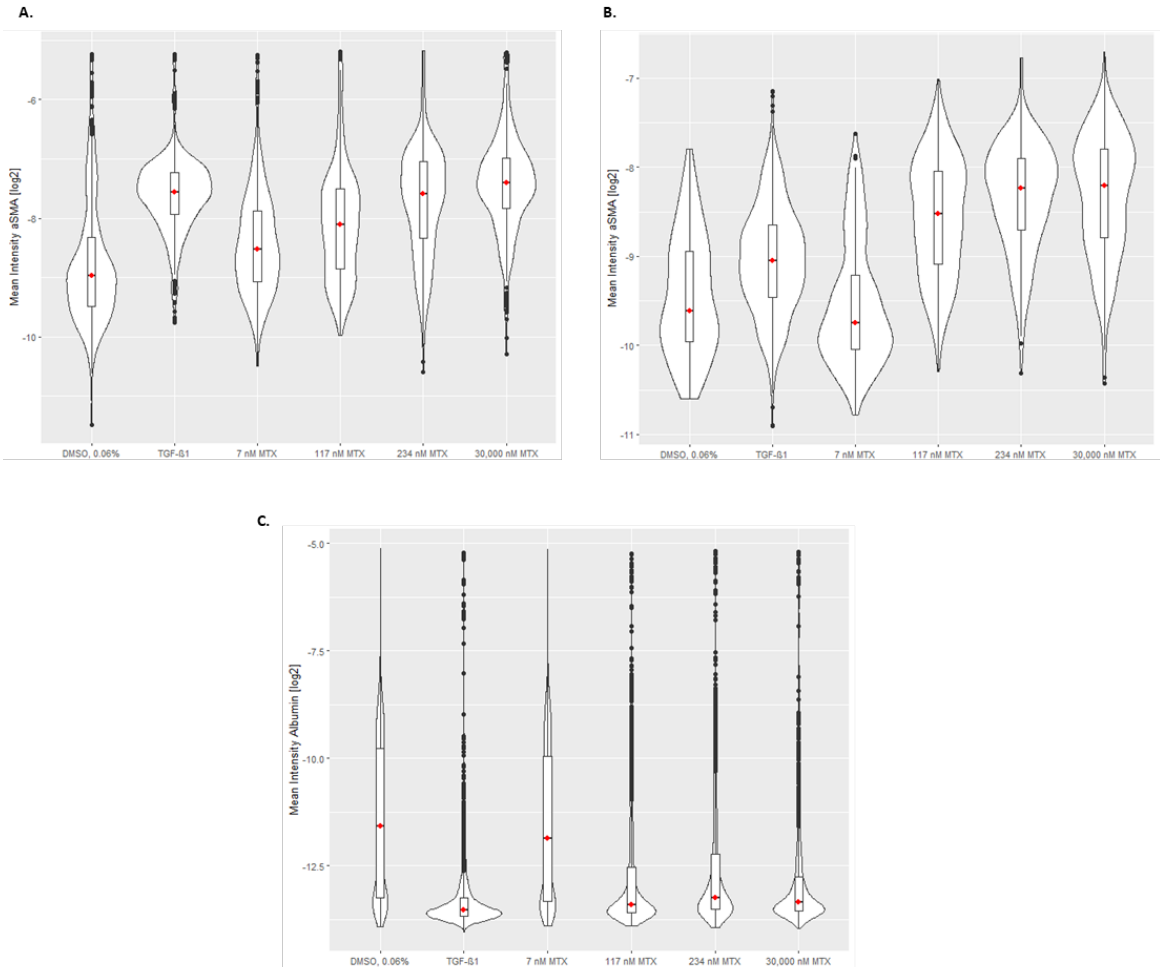

Figure S3. Intensity measurement of albumin and αSMA expression in mono- and co-cultures. hTERT-HSC (**A**) and co-cultures of HepaRG and hTERT-HSC (**B, C**) were exposed to varying concentrations of MTX and to the positive control TGF-β1 1 ng/mL for 7 days. Single cell analysis was performed using CellProfiler and RStudio (Figure 6). Activation of hTERT-HSC was confirmed by αSMA staining intensity analysis of the single cells. For the co-culture a threshold was applied to exclude albumin positive cells (**A, B**). Single cell analysis revealed decreasing levels of albumin staining intensity for all treatments (**C**). Median depicted in red (**A – C**).
